# Novel grape seed extract nanoparticles attenuate amikacin-induced nephrotoxicity in rats

**DOI:** 10.1186/s13568-023-01639-3

**Published:** 2023-11-20

**Authors:** Alyaa Farid, Dina Mohamed, Dina Mostafa, Rawan Tarek, Viola Sherif, Gehan Safwat

**Affiliations:** 1 Biotechnology Dep, Faculty of Science, Cairo University, Giza, Egypt; 2 Faculty of Biotechnology, October University for Modern Sciences and Arts (MSA), Giza, Egypt

**Keywords:** Amikacin, Grape seed, Nanoparticles, GC-MS, HPLC, Inflammation

## Abstract

Amikacin (AMK), an antibiotic, is prescribed for treating various bacterial diseases like urinary tract infections, encephalitis, asthma and joint infections. The most significant side effects, which affect 1 to 10% of consumers, are kidney injury and ototoxicity. Several studies discussed the role of grape seed extract (GSE) in renoprotection against AMK. The current study aimed to extract Muscat of Alexandria grape seeds followed by its characterization to determine its bioactive components and elements. GSE nanoparticles was prepared and tested, in vitro, to determine its safety for the in vivo experiment. Experimental groups were control group I, AMK group II, GSE (50 mg/kg)-AMK group III, GSE (100 mg/kg)-AMK group IV, GSE NPs (25 mg/kg)-AMK group V and GSE NPs (50 mg/kg)-AMK group VI. Groups 2–6 received 100 mg/kg/day of AMK by intramuscular injection for two weeks for the induction of experimental nephrotoxicity. Groups 3–6 received daily doses of GSE or GSE NPs by oral gavage, concurrently, with AMK for two weeks. GSE was rich in polyphenol compounds like proanthocyanidins, phenolic acids like gallic and egallic acids, catechine and epicatechine. GSE NPs have a smooth surface and a size that ranged from 40 to 70 nm; and have an anti-oxidant, anti-inflammatory, anti-cytotoxic and anti-microbial in vitro effects. It reduced oxidative stress and inflammation that followed AMK administration; and attenuated the AMK-induced nephrotoxicity. GSE NPs were safe to be used in vivo as a renoprotective agent against AMK; where, it reduced the oxidative stress and inflammation.

## Key message

Grape seed extract was rich in polyphenol compounds like proanthocyanidins.

Grape seed extract nanoparticles have a size that ranged from 40 to 70 nm.

Nanoparticles were safe to be used in vivo as a renoprotective agent against AMK.

## Introduction

Chronic changes in kidney-based shape, function, or both that have an impact on a person’s health are referred to as kidney disease (Zoccali et al. 2017). Cyst, tumor, malformation, and atrophy are a few examples of anatomical problems that are visible on imaging. Kidney failure, however, can cause oedema, hypertension, changes in urinary production or quality, and growth retardation in children. These changes are most frequently identified by elevated serum creatinine level and/or blood urea nitrogen (BUN). Regardless of the underlying medical condition, renal fibrosis is the most typical pathological symptom of kidney failure (Romagnani et al. 2017). Both the progression of kidney disease and its associated consequences are significantly influenced by the oxidative stress and inflammation (Massy and Nguyen-Khoa 2002). Renal cell failure is a consequence of oxidative stress, which is caused by a mismatch between the overproduction of reactive oxygen species (ROS) and the antioxidant enzymes deficiency (Wallace 2005).

In clinical terms, aminoglycoside-induced nephrotoxicity appears as nonoliguric renal failure, with a gradual increase in serum creatinine level and a hypoosmolar urine production appearing after a few days of therapy (Mingeot-Leclercq and Tulkens 1999). Frequent use of aminoglycoside drugs may result in chronic renal disease by damaging the kidneys; either at the level of the tubules (causing changed excretion of ions) or the glomerulus (causing reduced glomerular filtration rate) (Prayle et al. 2010). Aminoglycosides induce kidney damage because a minor but significant part of the given dosage (nearly 5%) remains trapped in the epithelial cells coating the proximal tubules (S1 and S2 regions) following glomerular filtration (Sandoval et al. 1998). Aminoglycosides are accumulated in the lysosomes, endosomal vacuoles and golgi apparatus in these cells. The release of significant amounts of trapped aminoglycoside from lysosomes may, under certain circumstances, result in the simultaneous emergence of a variety of metabolic alterations that are capable of inducing cell death (Mingeot-Leclercq and Tulkens 1999).

There are numerous studies looking for methods to decrease amikacin (AMK), as an aminoglycoside, nephrotoxicity. Potential strategies include suppressing the signaling between cells that causes apoptosis, decreasing the production of ROS, preventing tubular absorption, and improving the kidney’s capacity for regeneration. For many years, chronic kidney disease (CKD) patients have used complementary and alternative therapies, with apparently varying degrees of success in enhancing the outcomes (Zhong et al. 2013). While these treatments are most likely used in conjunction with traditional medicine in Western nations, they are the only form of treatment for CKD patients in nations like China, Asian and African nations (Gobe and Wojcikowski 2019).

Dietary supplementary grape seed extract (GSE) is available as a powder, beverage, pills and tablet (Nassiri-Asl and Hosseinzadeh 2016). It is produced from the seeds of various vines (*Vitis vinifera*), after being removed, dried, and grounded (Perumalla and Hettiarachchy 2011). Surprisingly, grapes’ seeds contain the largest proportion of the fruit’s overall polyphenols. GSE is made up of a variety of vitamins and 3% minerals, 35% fibres, 13% fats, 11% proteins, 7% water; and a heterogeneous combination of polyphenolic substances, including 5 to 30% monomers, 17 to 63% oligomers and 11 to 39% polymers (Ma and Zhang 2017). Proanthocyanidins have been identified chemically as the primary polyphenolic components of GSE (Mandic et al. 2008). It is well known that proanthocyanidins have anti-inflammatory, anti-oxidant, anti-hypertensive, anti-platelet, antithrombotic and cholesterol-lowering properties (Cos et al. 2004). It’s interesting to note that under in vitro circumstances, antioxidant ability of GSE was found to be more powerful than those of vitamins C and E (Bagchi et al. 1997). GSE protects against high fat diet (HFD)-induced obesity and lipotoxicity in rats by protecting the heart, liver, the brain and kidney (Charradi et al. 2011, 2012, 2013, 2014). In addition, high doses of GSE have been demonstrated to reduce renal injury in diabetic animals through their antioxidant and anti-inflammatory actions (Bao et al. 2015); and protect against the nephrotoxicity caused by arsenic, cisplatin, amikacin, and cyclospor (Ulusoy et al. 2012; Gao et al. 2014; Zhang et al. 2014; Bao et al. 2015).

Despite the fact that GSE is frequently found in nutritional products, excessive consumption of it may be unfavorable. The adverse effects of GSE include an increased chance of haemorrhage; additionally, GSE might result in nausea, dizziness, indigestion, diarrhea, headache, dry scalp, rash, cough and sore throat (Berry et al. 2016). In some instances, it can cause allergic responses, affect how well some medicines work (particularly blood thinners) and interact with other drugs (broken down by the liver) (Berry et al. 2016). Bijak et al. (2019) reported the possible impact of GSE on clotting mechanisms; they strongly imply that GSE may be a hopeful nutraceutical for the avoidance of coronary thrombotic events brought on by various mechanisms. The clotting process in human plasma was modulated by GSE, according to in vitro research of Bijak et al. (2011). Where, the coagulation time (PTT and PT) and thrombin-induced plasma polymerization were both prolonged by the effect of GSE. Additionally, the primary human clotting enzyme, thrombin, is severely inhibited by this extract’s proteolytic and amidolytic activity (Bijak et al. 2013). Therefore, when GSE combined with other blood thinners like aspirin or warfarin, it may function as a blood thinner and raise the chance of bleeding. Although there were a case report of GSE-induced contact dermatitis, GSE had no impact and was used as the placebo control in a seven-month controlled trial of aromatherapy on alopecia (Krogsrud and Larsen 1992; Hay et al. 1998). Moreover, some GSE formulations contain up to 8% benzethonium chloride, a disinfectant that is classified as a class 2 toxin due to its teratogenicity and caustic properties (Takeoka et al. 2001; Berry et al. 2016). Some GSE products contain tricolsan, a preservative that the Environmental Protection Agency classifies it as a pesticide. Tricolsan is connected to a variety of bis-phenyl cholorphenol and bis-phenyl polychlorinated substances that are known to have negative health impacts (Caldecott 2005). Following the use of GSE, a 49-year-old man was described by Berry et al. (2016) as having persistent sickness, diarrhea, nausea, and acute fatigue. No reasons were found after a comprehensive investigation by a medical professional using imaging, procedures, or therapeutic methods. After GSE was stopped, symptoms disappeared, and five years later the patient was still symptom-free.

Nanoparticles (NPs) have a number of benefits, including a long lifespan, the ability to incorporate water-friendly and water-resistant compounds, and the ability to be administered via a variety of methods (including inhalation and oral application); in addition to, the controlled drug release. These characteristics of NPs increase medication bioavailability, decrease doses’ concentration and frequency. Therefore, the current study aimed to: 1- extract Muscat of Alexandria grape seeds, 2- characterization of GSE to determine its bioactive components and elements, 3- synthesize and characterization of GSE NPs, 4- test the safety of GSE NPs in vitro and 5- evaluate their effects on amikacin-induced nephrotoxicity in rats.

## Materials and methods

### Preparation of GSE

Muscat of Alexandria grape seeds were separated, air-dried in the shade at room temperature, and then ground into a powder. Powdered grape seeds (500 g) were combined with 160 ml of water and 1440 ml of ethyl alcohol, according to Farid et al. (2022). GSE was filtered and left under reduced pressure (40^o^C) to allow alcohol evaporation.

### Characterization of GSE

#### Gas chromatography–mass spectrometry (GC-MS) technique

The chemical constituents of GSE was examined using a Thermo Scientific Trace GC1310-ISQ mass spectrometer and a TG-5MS direct capillary column (30 m x 0.25 mm x 0.25 m film thickness). The column oven’s temperature was initially fixed at 50 °C, then increased by five degrees Celsius per minute to 230 °C and maintained for 2 min, and then raised to 290 °C by thirty degrees Celsius per minute and sustained for 2 min. A constant flow rate of helium (1 ml/min) was used as the carrier gas, and the injector and MS transfer line temperatures were maintained at 250 and 260 °C, respectively. A three-minute solvent delay was automatically introduced into diluted samples (1 µl) by the autosampler when used with a gas chromatography (split mode).

#### High performance liquid chromatography (HPLC) technique

HPLC analysis was carried out using an Agilent 1260 series with an Eclipse C18 column (4.6 mm x 250 mm i.d., 5 m) at 40 ^o^C. The mobile phase’s components were water (A) and 0.05% trifluoroacetic acid/acetonitrile (B) at 0.9 ml/min flow rate. The mobile phase was successively designed to run for the following durations: 0 min (82%A), 0 to 5 min (80%A), 5 to 8 min (60%), 8 to 12 min (82%A), 15 to 16 min (82%A), and 16 to 20 min (82%A). 5 µl of GSE was injected and the results was detected at 280 nm.

#### Inductively coupled plasma (ICP) technique

The elements found in grape seeds were identified by ICP using the ashing method. In a crucible, dried grape seeds (2.5 g) were placed and progressively heated to 105, 150, 270 ^o^C, and then maintained at 570 ^o^C for four hours. After the samples were burnt to ashes and dried, five ml of HCl, HNO_3_, and H_2_O_2_ (1:1:3 v/v mixture) was added twice. After the residue had been dissolved in 1 M HNO_3_ (10 ml), it was centrifuged. ICP was then used to determine the concentration of each element in grape seeds.

#### Preparation of GSE NPs

GSE powder (5 g) was added to 50 ml HCl (30%) and stirred for 60 min (at 30° C and 3000 rpm), followed by the addition of 100 ml distilled water. The solution was stirred for an additional two hours, followed by filtration to recover GSE NPs. Transmission electron microscope (TEM) was used to determine the size of prepared NPs, while scanning electron microscope (SEM) was used to observe the shape of NPs.

### Characterization of GSE NPs

#### In vitro coagulation activity of GSE NPs

The anticoagulant activity of GSE NPs was assessed by measuring the coagulation time in seconds with heparin serving as the reference. Following the manufacturer’s directions, prothrombin time (PT) and partial thromboplastin time (PTT) diagnostic tests were used. In brief, 100 µl of heparin or varying concentrations of GSE NPs (25, 50, and 75 µg/ml) were combined with 900 µl of rat plasma. The clotting time was determined after the procedure was performed four times at 37 °C.

#### In vitro anti-inflammatory (membrane stabilization) activity of GSE NPs

Red blood cells (RBCs) were prepared from heparinized rat blood by centrifugation at 2000 rpm for 25 min; then the pellet was dissolved in fresh saline. Different concentrations of GSE NPs (100, 200, 400, 600, 800, and 1000 µg/ml) were mixed, individually, with 5 ml of hypotonic solution like distilled water. The same doses of GSE NPs were mixed with 5 ml saline. Indomethacin (200 µg/ml) acted as a standard control. Prepared NPs solutions and indomethacin were added to the prepared RBCs suspension (0.1 ml), kept for an hour at 37 °C and centrifuged at 1500 rpm for 10 min. A spectrophotometer (at 540 nm) was used for estimating the quantity of released haemoglobin in the supernatant, and the formula hemolysis inhibition (%) = 1- [(ODb - ODa) / (ODc - ODa)] X 100 was used to determine the proportion of hemolysis inhibition. The symbols ODa, ODb, and ODc, respectively, stand for the substances’ absorbance in saline, water, and indomethacin.

#### The antioxidant activity of GSE NPs

1, 1-diphenyl-2-picryl hydrazyl (DPPH) was used to assess the antioxidant activity of the produced GSE NPs in comparison to that of ascorbic acid (control). Briefly, different concentration of GSE NPs (3.9, 7.8, 15.62, 31.25, 62.5, 125, 250, 500, and 1000 µg/ml) were mixed, individually, with one ml of a DPPH/ethanol solution, shaken and left for half an hour at room temperature. The DPPH scavenging activity (%) was determined by the formula: [(A0-A1)/A0] X 100; where, A1 indicated the sample absorbance and A0 indicated the control absorbance.

#### In vitro cytotoxicity activity of GSE NPs

10^5^ WI38 and HCT116 cells were cultures on tissue culture plates for 24 h at 37 °C to allow the development of cell monolayers. RPMI medium was supplemented with GSE NPs (31.25, 62.5, 125, 250, 500, and 1000 µg/ml); where, cultured WI38 and HCT116 cells received 100 µl/well of diluted NPs followed by a 24-hour incubation period. After incubation, 20 µl/well of MTT (3-(4,5-dimethylthiazol-2-yl)-2-5-diphenyltetrazolium bromide) were added; and plates were incubated for 4 h at 37 °C and 5% CO_2_. DMSO was added to the plates to dissolve the formazan that had developed and the absorbance was recorded at 560 nm.

#### The antimicrobial activity (disk diffusion method)

The microbial strains [*Bacillus subtilis* (ATCC 6633), *Staphylococcus aureus* (ATCC 6538), *Escherichia coli* (ATCC 8739), *Pseudomonas aeruginosa* (ATCC 90,274), *Mucor* and *Candida albicans* (ATCC 10,221)] were purchased from Microbiological Resources Center (Cairo MIRCEN); and were cultured in Mueller Hinton broth at 37 °C for 24 h with 200 rpm agitation. By the aid of a clean cotton swab, the microbial strains were applied to the Mueller-Hinton agar. GSE and GSE NPs (10 µl) were loaded, independently, onto the discs. After 24 h of incubation at 37 °C, the inhibitory zone was measured.

#### Experimental design

Forty eight male Sprague dawely rats (160–180 g) were divided into six groups: control group I, AMK group II, GSE (50 mg/kg)-AMK group III, GSE (100 mg/kg)-AMK group IV, GSE NPs (25 mg/kg)-AMK group V and GSE NPs (50 mg/kg)-AMK group VI. Groups 2–6 received 100 mg/kg/day of AMK (Sigma Aldrich, USA) by intramuscular injection for two weeks for the induction of experimental nephrotoxicity (Batoo et al. 2018). Groups 3–6 received daily doses of GSE or GSE NPs by oral gavage, concurrently, with AMK for two weeks. Rats were obtained from the animal house colony, National Research Centre, Cairo, Egypt; and housed under standard experimental condition (22 ± 2^o^C, 12 h dark/light cycle and 50 ± 2% humidity) for one week for acclimatization. Animals received standard rat’s diet and water *ad libitum*, throughout the experimental time; and all experimental procedures were accomplished according to the ARRIVE guidelines for the reporting of animal experiments. Rats were anaesthetized with sodium pentobarbital (50 mg/kg) at the end of the research, and blood was drawn by heart puncture and then centrifuged to separate the serum. Aliquots of sera were kept at 80 °C. All experimental groups’ kidney samples were collected and were split into two parts: one for the histological analysis and the other for the biochemical analysis. For the preparation of kidney tissue homogenates, one g was homogenised in 5 ml of cold Tris-HCl solution (10 mmol, pH = 7.4) and centrifuged for 15 min at 4 °C and 2000 rpm. The protein concentration of the supernatant was determined (Farid et al. 2022).

#### Biochemical analysis

Blood urea nitrogen (BUN), creatinine and uric acid were measured by rat’s ELISA kits (MBS2611086, MBS3809095 and MBS7606443, respectively; MyBioSource, USA) in serum samples. Albumin was determined by rat’s ELISA kit (ab108789, abcam, UK) in urine samples; where, animals were, separately, placed in metabolic cages (with free access to water) overnight for the collection of urine samples (Wiedmeyer and Royal 2010). Oxidative stress was evaluated by measuring malondialdehyde (MDA) and superoxide dismutase (SOD) in the kidney tissue homogenates by colorimetric assay kits (ab118970 and ab65354, respectively; abcam, UK). Pro-inflammatory cytokines (TNF-α, IL-4 and IL-6) were determined in kidney tissue homogenates (ab100785, ab100770 and ab234570, respectively; abcam, UK). All procedures of ELISA were performed according to the manufacturer’s instructions and precautions of Hegazy et al. (2015) and Farid et al. (2023).

#### Histopathological examination

Kidney samples from the various study groups were fixed in buffered formalin (10%), then dehydrated, cleared with xylene, and embedded in paraffin wax. tissue blocks were cut into sections with 4 μm thickness, stained with hematoxylin and eosin (H&E) and mounted. The examination was carried out by a skilled blinded pathologist who was unaware of the design of the experiment.

### Statistical analysis

The results were presented as mean ± SE; where, ANOVA test was used to analyse the data and the Tukey post hoc test was used to compare the results. At p < 0.05, values were regarded significant.

## Results

### Results of GC-MS analysis

Alcohols of terpenes like geraniol, borneol, linalool, and -terpeniol were detected in GSE by GC-MS. Additionally, phenolic acids such as gallic and egallic acids (typical components of grape seed) were discovered, along with aldehydes such as the benzoic aldehyde (characteristic of vegetables seeds). The findings revealed the existence of several classes of flavonoids, including flavanols (catechine and epicatechine), flavonols (Kaempferol, Myricetin and quercetin). Stilbens was presented by resveratrol (Table 1).

**Table 1:** Identified compounds in GSE by GC-MS analysis

Compound	Formula	Retention time (min)	Percent (%)
**Xanthosine**	C_10_H_12_N_4_O_6_	6.67	2.6
**Palmitic acid, ethyl ester**	C_18_H_36_O_2_	10.12	3.7
**Benzoic aldehyde**	C6H5CHO	10.4	1.7
**Oleic acid, methyl ester**	C_19_H_36_O_2_	11.68	6.7
**linoleic acid, ethyl ester**	C_20_H_36_O_2_	12.1	5.9
**d-Mannose**	C_6_H_12_O_6_	13.2	1.1
**Camphene**	C_10_H_16_	13.9	0.5
**Geraniol**	C_10_H_18_O	16.6	6.6
**Linolinic acid**	C_18_H_30_O_2_	16.8	0.3
**Hexadecanoic acid**	C_16_H_32_O_2_	18.3	5.3
**Borneol**	C_10_H_18_O	18.4	0.9
**Linalool**	C_10_H_18_O	18.6	2.3
**Ellagic acid**	C_14_H_6_O_8_	18.7	3.7
**Linoleic acid**	C_18_H_32_O_2_	18.8	4.1
**Gallic acid**	C_7_H_6_O_5_	18.9	4.6
**Resveratrol**	C_14_H_12_O_3_	19.2	2.6
**α-caryophyllene**	C_15_H_24_	20.9	1.3
**Quercetin**	C_15_H_10_O_7_	22.8	6.2
**α –terpeniol**	C_10_H_18_O	23.9	1.1
**Catechine**	C_15_H_14_O_6_	24.2	2.4
**Kaempferol**	C_15_H_10_O_6_	24.5	7.5
**Epicatechine**	C_15_H_14_O_6_	24.6	2.3
**Myricetin**	C_15_H_10_O_8_	24.8	6.7

### Results of HPLC analysis

According to the results of HPLC, the major compounds found in GSE were gallic acid, coffeic acid, catechin and epicatechin (1.7, 1.3, 1.9 and 1.5 mg/100 mg, respectively). In addition to a high number of procyanidins (monomer, dimer and polymers) (Table 2).

**Table 2:** Identified compounds in GSE by HPLC analysis

Compounds Name	Retention time (min)	Conc. (mg/100 mg )
**Gallic acid**	8.1	1.7
**Coffeic acid**	9.3	1.3
**Procyanidin dimer-B3**	30.1	0.6
**Procyanidin dimer-B1**	33.2	1.4
**Catechin**	35.4	1.9
**Procyanidin dimer-B4**	45.3	0.8
**Procyanidin dimer-B2**	52.4	1.8
**Epicatechin**	62.4	1.5
**Procyanidin dimer-B7**	73.2	0.4
**Procyanidin trimer-C**	75.4	0.7
**Procyanidin tertramer**	80.3	0.3
**Procyanidin pentamer**	82.6	0.3
**Procyanidin hexamer**	86.4	0.1
**Epicatechin-galat**	91.4	0.2

### Elemental analysis by ICP technique

From the ICP result, the highest elements’ concentration was of calcium followed by potassium (6.2 and 4.1 mg/g, respectively). Other elements were found in GSE like zinc, copper, magnesium and manganese (Table 3).

**Table 3:** Elements’ concentration (mg/g) in GSE

Element	Concentration (mg/g)
**Sodium**	0.1
**Zinc**	0.03
**Copper**	0.002
**Potassium**	4.1
**Magnesium**	0.8
**Manganese**	0.04
**Calcium**	6.2
**Iron**	0.2

### Physical characterization

**Fig. 1:**
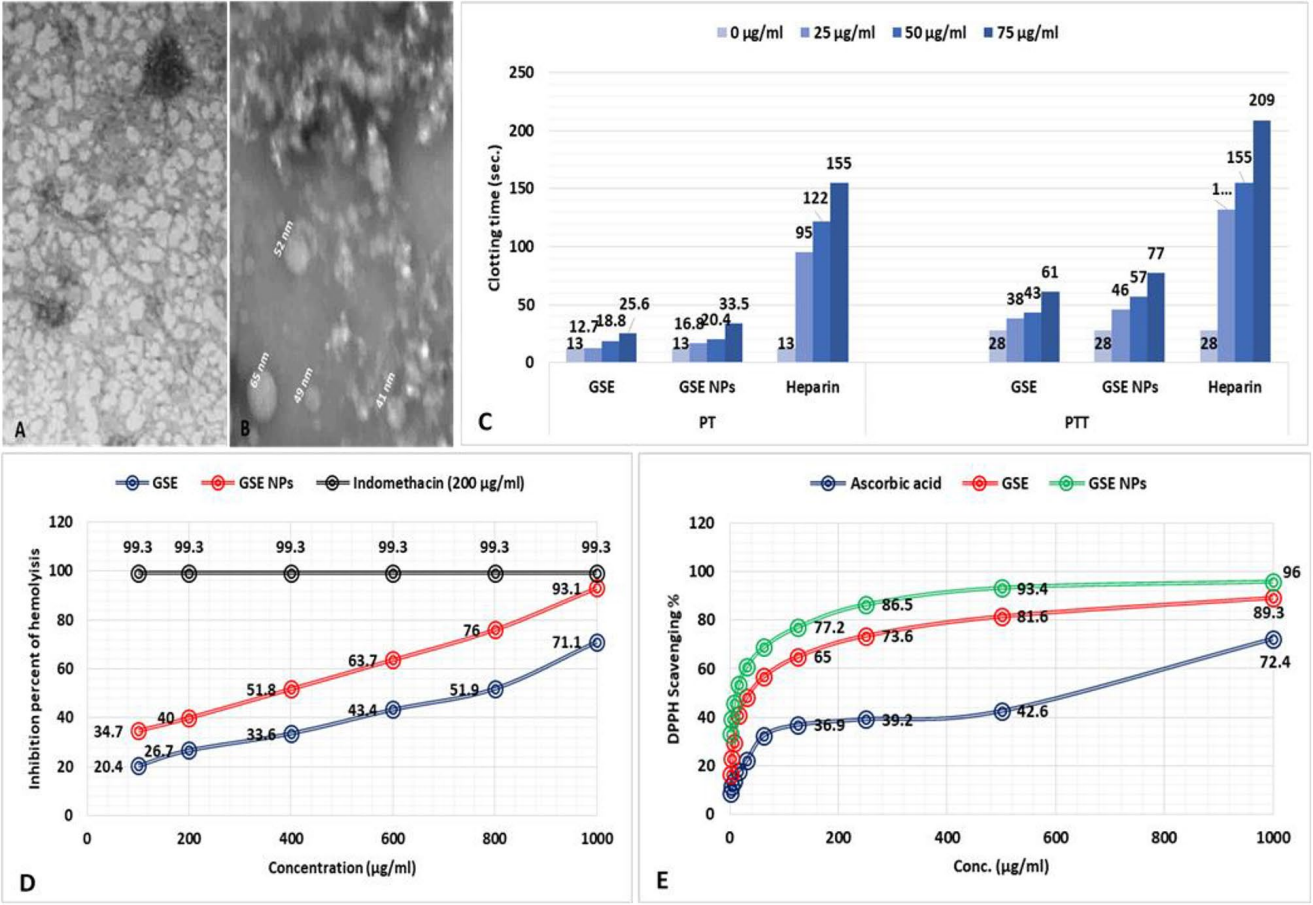
**A**] SEM image of GSE NPs, **B**] TEM image of GSE NPs, **C**] The anticoagulant activity of GSE and GSE NPs (25, 50 and 75 µg/ml), **D**] The in vitro anti-inflammatory effect of GSE and GSE NPs (100, 200, 400, 600, 800 and 1000 µg/ml) and **E**] DPPH scavenging % of GSE and GSE NPs (1000, 500, 250, 125, 62.5 and 31.25 µg/ml)

GSE NPs have a size that ranged from 40 to 70 nm (Fig. 1B) with a smooth surface (Fig. 1A).

### The in vitro anticoagulant effect of GSE and GSE NPs

The clotting time was determined by measuring both of PT and PTT at 25, 50 and 75 µg/ml of GSE or GSE NPs. GSE achieved a PT of 12.7, 18.8 and 25.6 s; while GSE NPs achieved a PT of 16.8, 20.4 and 33.5 s for 25, 50 and 75 µg/ml, respectively. Also, a direct relation between the concentration of GSE or GSE NPs and the PTT was observed (Fig. 1C). However, the PT and PTT of GSE NPs was higher than those of GSE at all used concentration.

### The in vitro anti-inflammatory effect of GSE and GSE NPs

In comparison to indomethacin (200 µg/ml), GSE NPs inhibited hemolysis of red blood cells (RBCs) by 93.1% followed by GSE (71.1%) at 1000 µg/ml. however, hemolysis inhibition increased in a dose dependent manner for both of GSE and GSE NPs (Fig. 1D).

### The in vitro antioxidant effect of GSE and GSE NPs

The GSE NPs showed the highest DPPH scavenging % at all tested concentration (1000, 500, 125, 62.5 and 31.25 µg/ml) when compared to GSE and ascorbic acid (control). Moreover, the antioxidant activity of GSE and GSE NPs was increasing with the elevation in concentration; where, 96 and 89.3% were achieved with 1000 µg/ml of GSE NPs and GSE, respectively (Fig. 1E).

### Cytotoxicity (MTT) assay results

**Fig. 2:**
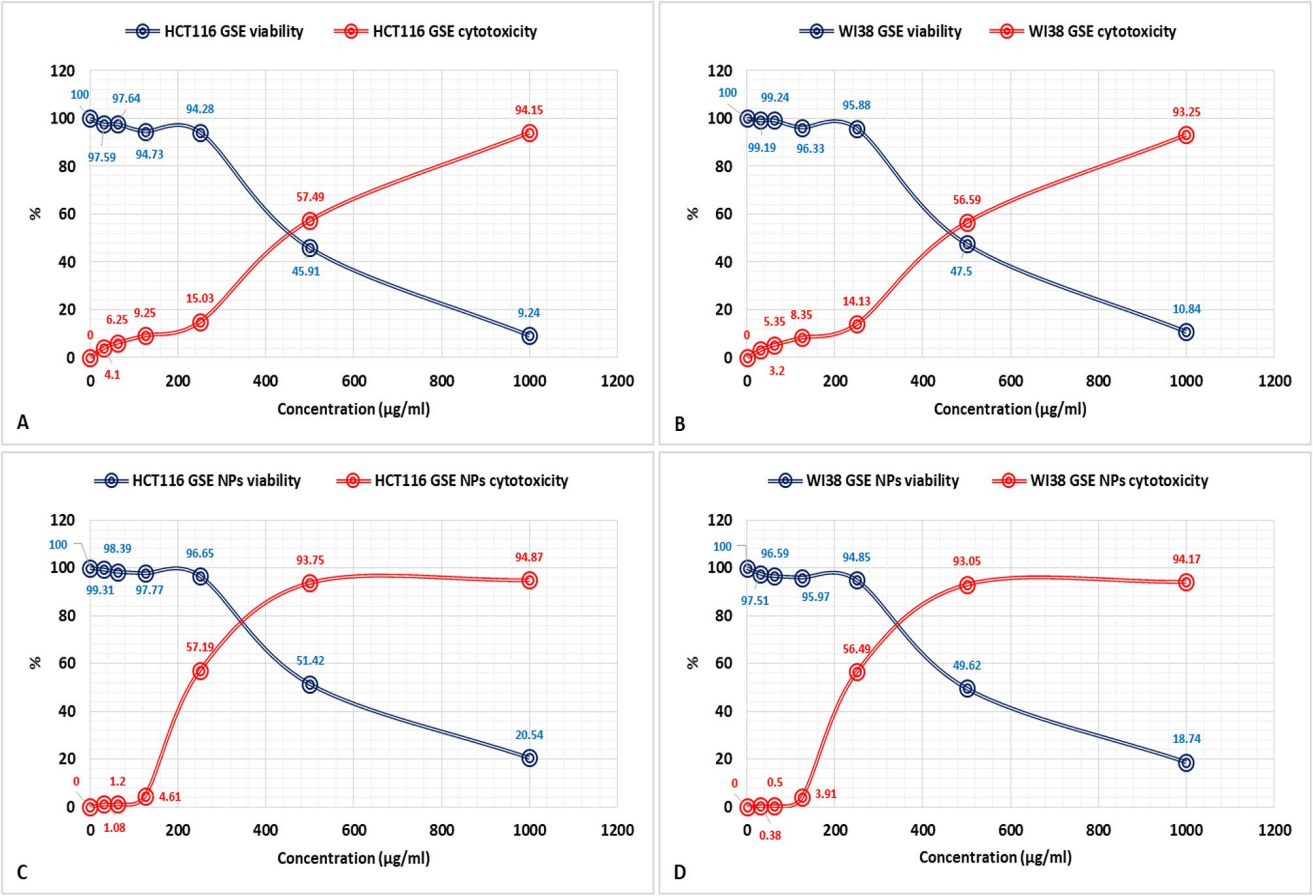
The results of cytotoxicity (MTT) assay of GSE (**A** and **B**) and GSE NPs (**C** and **D**), against WI38 and HCT116, at a concentration of 1000, 500, 250, 125, 62.5, 31.25 and zero µg/ml

A decrease in viability of WI38 and HCT116 was observed with increasing the concentration of GSE or GSE NPs. However, GSE NPs have an anti-cytotoxic effect against HCT116 more than GSE; where a viability % 99.31 and 97.59% were noticed for GSE NPs and GSE, respectively, at 31.25 µg/ml (Fig. 2). The opposite was observed with WI38, where a viability of 97.51 and 99.19% were observed for WI38 and HCT116, respectively, at the same concentration (31.25 µg/ml). Overall, a direct relation was documented between cytotoxicity % and concentration of GSE or GSE NPs; and an inverse relation between viability % and concentration.

### The in vitro antimicrobial effect of GSE and GSE NPs

**Fig. 3:**
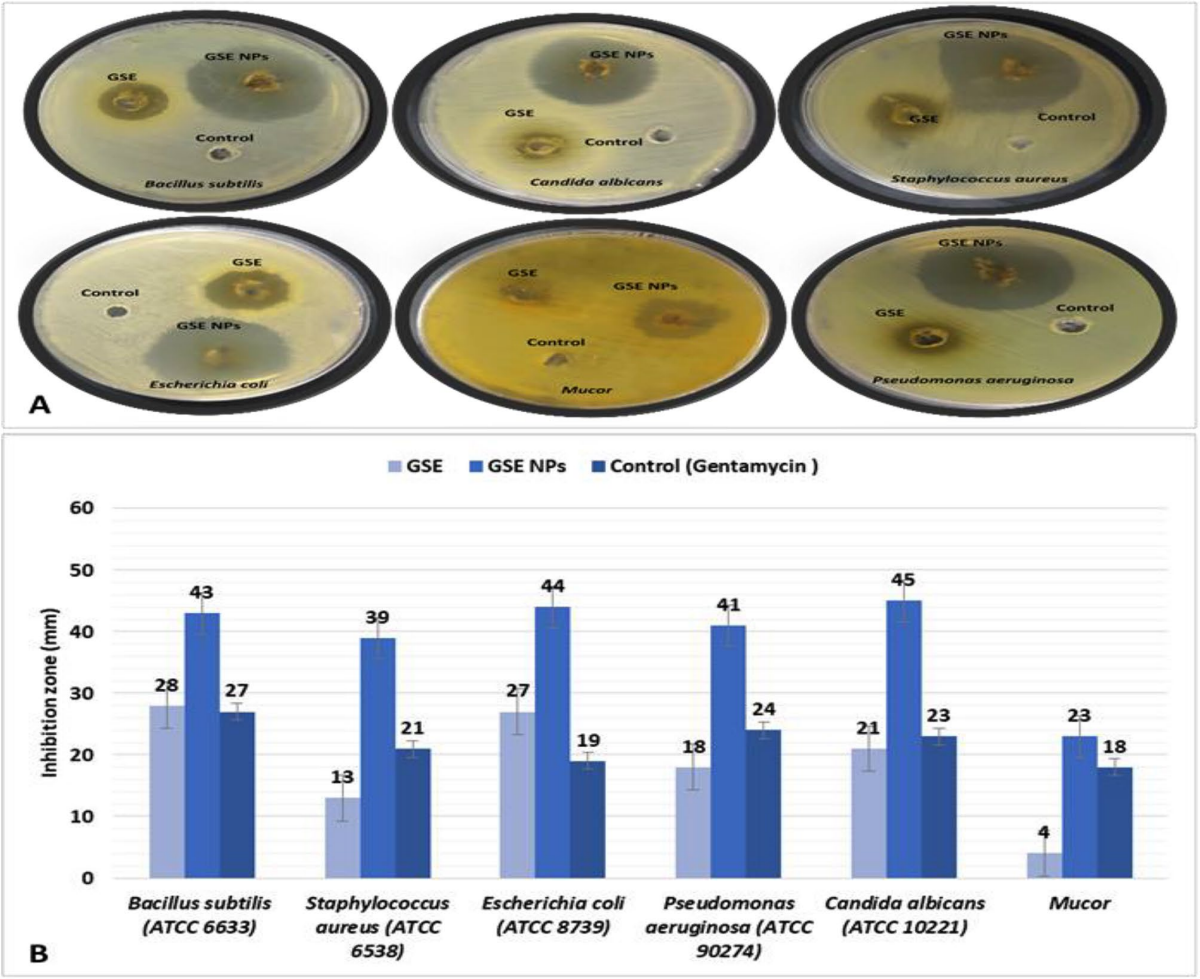
**A**] Photos of agar plates to show the antimicrobial activity of GSE and GSE NPs and **B**] The antimicrobial activity of GSE or GSE NPs against *Bacillus subtilis*, *Staphylococcus aureus*, *Escherichia coli*, *Pseudomonas aeruginosa*, *Mucor* and *Candida albicans*

The tested GSE NPs showed a strong antimicrobial activity, against all used bacterial and fungal strains, more than that of GSE (Fig. 3A). Where, GSE NPs led to inhibition zones of 43, 39, 44, 41, 45 and 23 mm with *Bacillus subtilis*, *Staphylococcus aureus*, *Escherichia coli*, *Pseudomonas aeruginosa*, *Candida albicans* and *Mucor*, respectively, when compared to GSE (28, 13, 27, 18, 21 and 4, respectively) (Fig. 3B).

### Kidney function

**Fig. 4:**
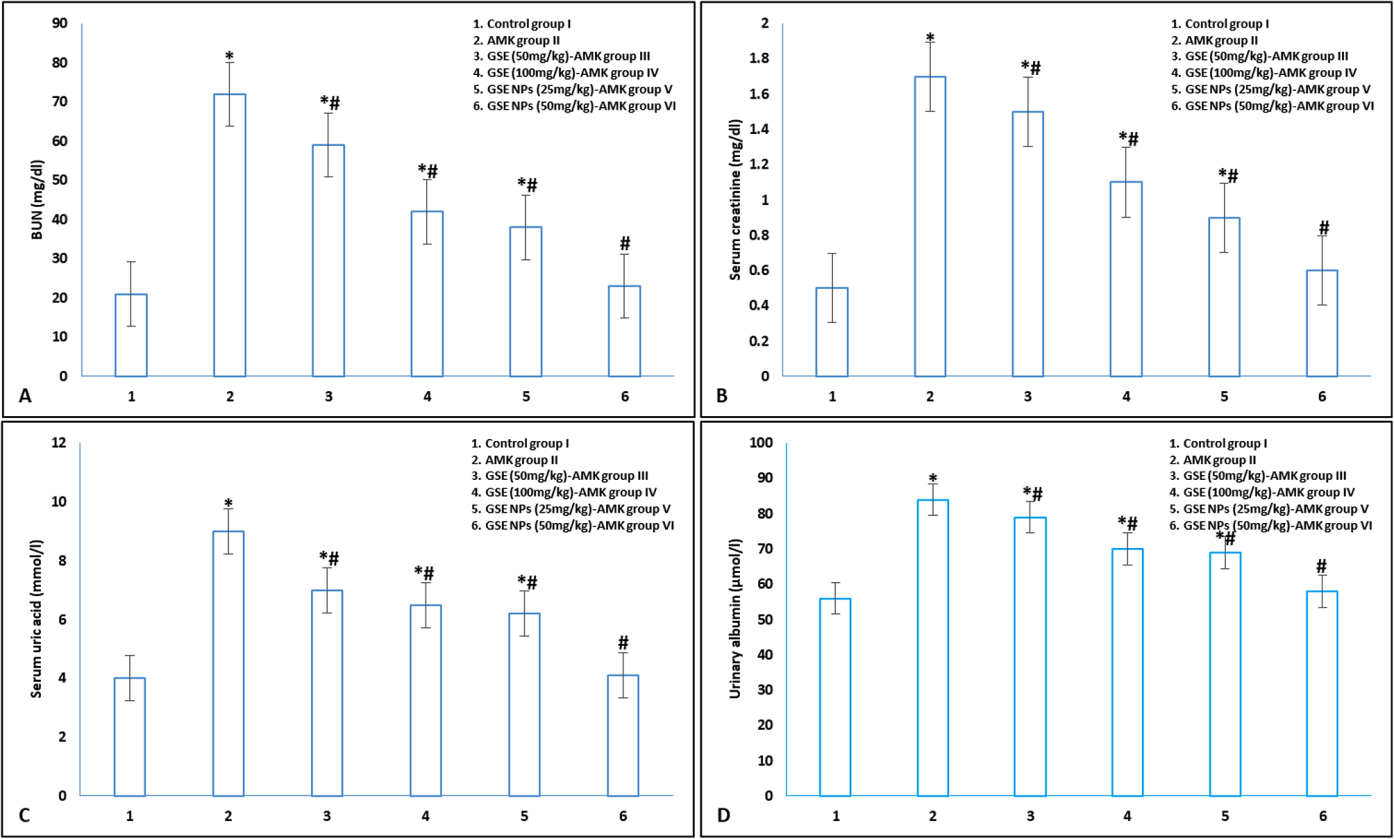
Kidney function parameters **A**] BUN, **B**] serum creatinine, **C**] serum uric acid and **D**] urinary albumin in different experimental groups. * represents significance (p < 0.001) with control group I and # represents significance (p < 0.001) with untreated AMK administrated group II

AMK administration significantly elevated BUN, urinary albumin, serum creatinine and uric acid in comparison to control group I (Fig. 4). GSE (either 50 or 100 mg/kg) and GSE NPs (25 mg/kg) administration significantly reduced kidney function parameters when compared to untreated AMK administrated group II. Group VI, administrated with 50 mg/kg GSE NPs, showed no significant difference in all measured parameters when compared to control group I.

### Oxidative stress in kidney tissue homogenates

**Fig. 5:**
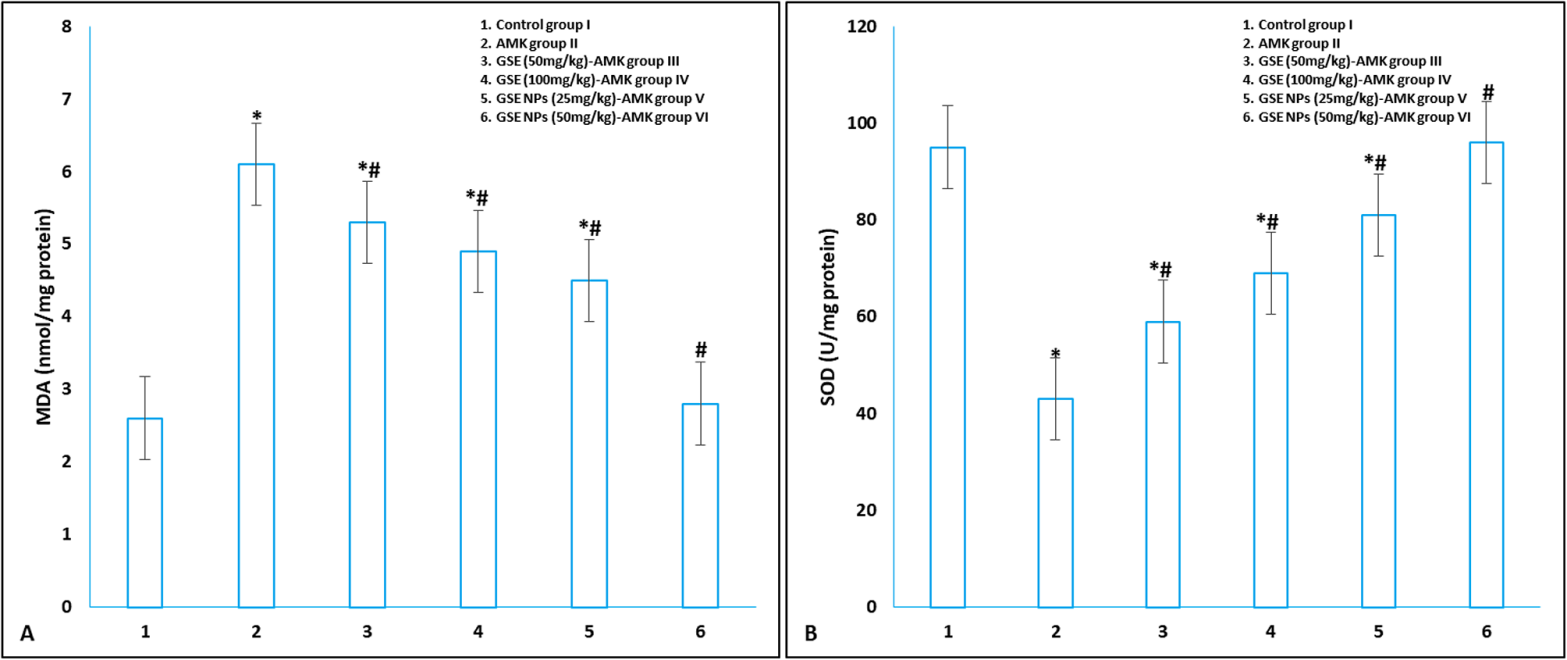
Oxidative stress parameters **A**] MDA and **B**] SOD in different experimental groups. * represents significance (p < 0.001) with control group I and # represents significance (p < 0.001) with untreated AMK administrated group II

MDA level showed a significant increase in response to AMK administration, in group II, when compared to control group I. on the other hand, SOD level was significantly reduced up on AMK administration in group II; and began to rise after GSE and GSE NPs administration. However, GSE NPs (50 mg/kg) administration succeeded in returning MDA and SOD levels to their normal range in comparison to control group I (Fig. 5).

### Pro-inflammatory cytokines in kidney tissue homogenates

**Fig. 6:**
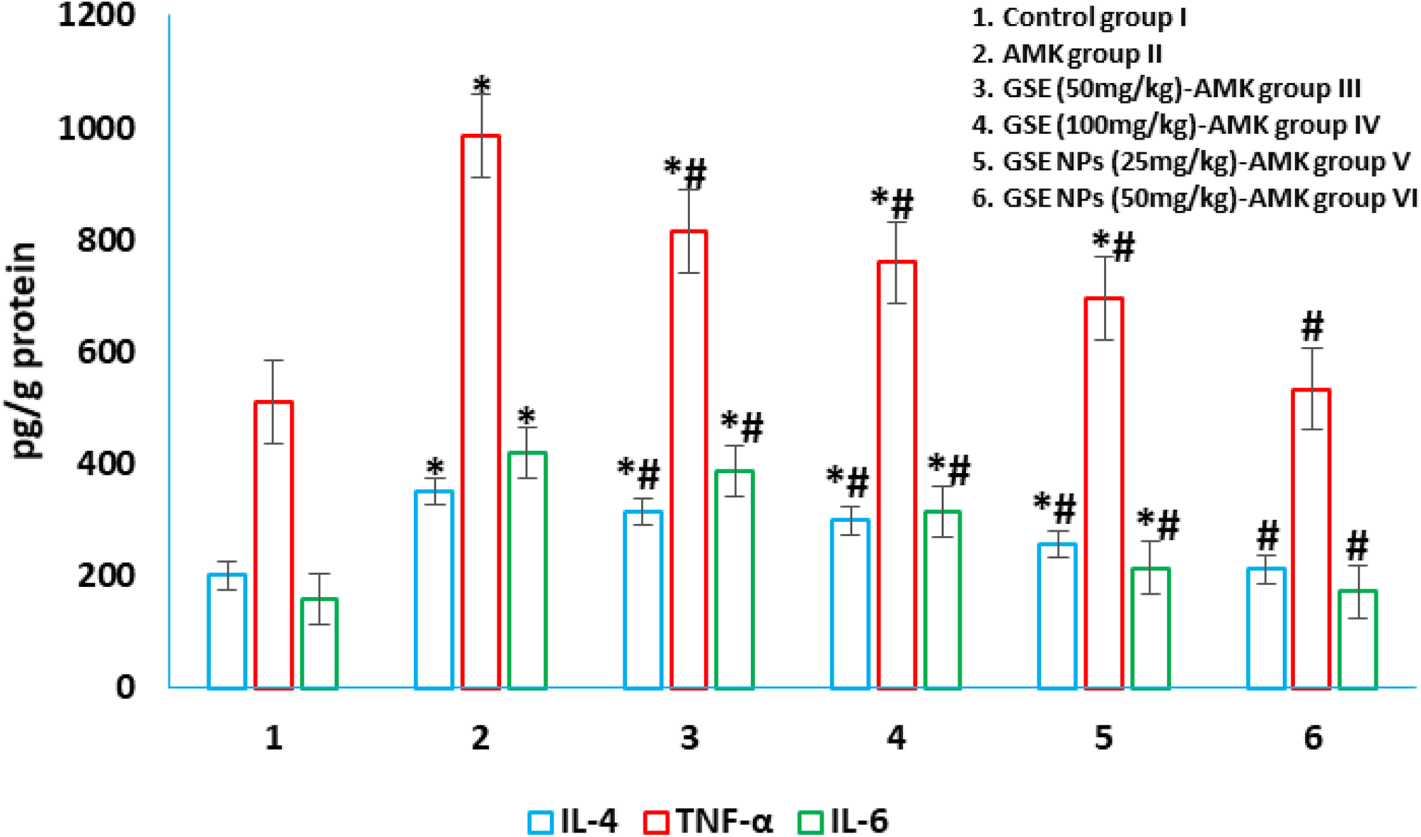
Pro-inflammatory cytokines (TNF-α, IL-4 and IL-6) in different experimental groups. * represents significance (p < 0.001) with control group I and # represents significance (p < 0.001) with untreated AMK administrated group II

An elevation in pro-inflammatory cytokines (TNF-α, IL-4 and IL-6) was observed in AMK administered untreated group II. Group VI, treated with 50 mg/kg, showed no significance difference in cytokines levels when compared to control group I. although GSE (either 50 or 100 mg/kg) administration decreased the cytokines levels when compared to those of untreated group I; their levels remained higher than those of control group I (Fig. 6).

### Histopathological results

**Fig. 7:**
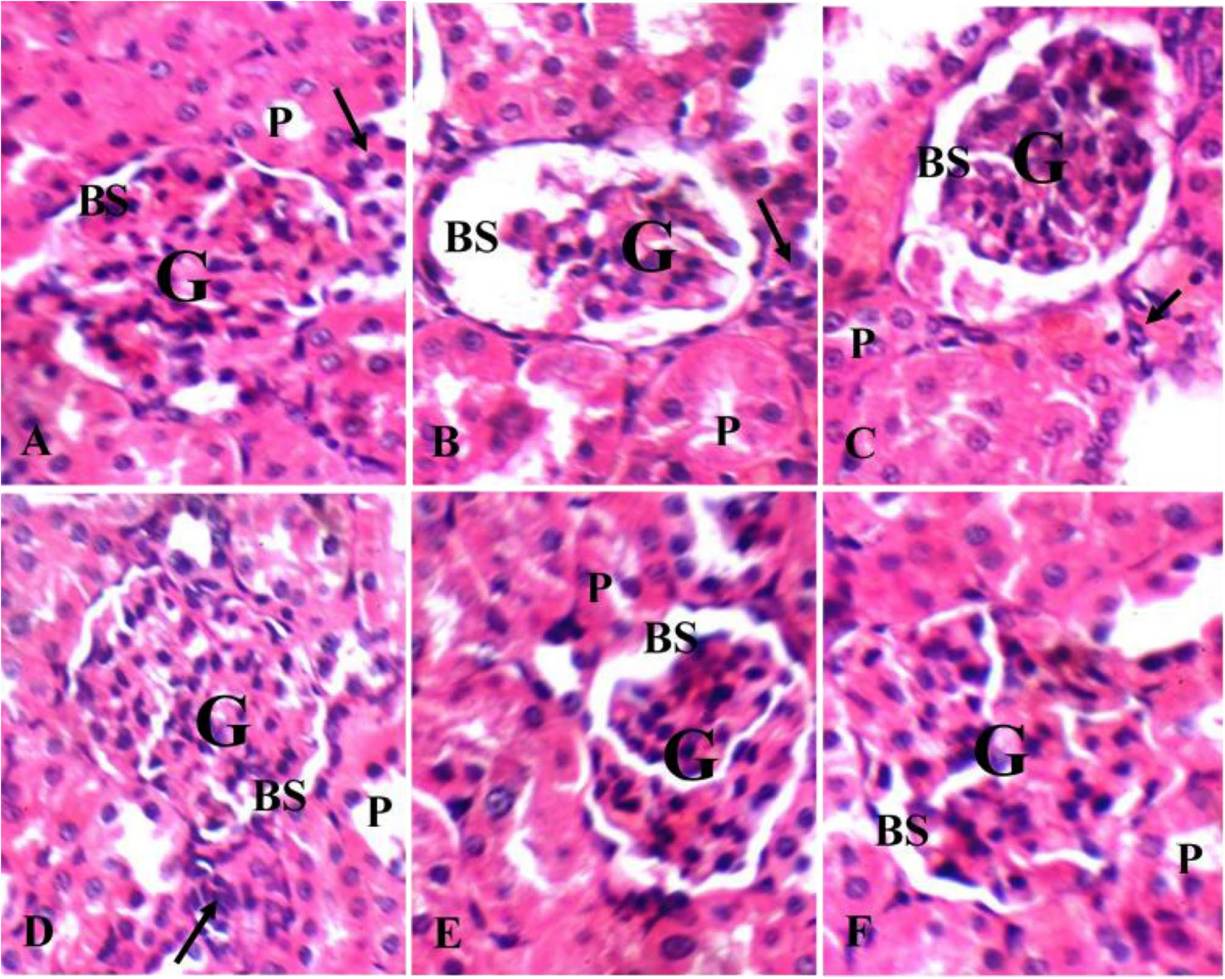
Haematoxylin and eosin rat’s kidney sections showing (**A**) and (**F**) average sized glomeruli (**G**) with average Bowman’s spaces (**BS**), proximal tubules (**P**) with average epithelial lining, and average interstitium in control group I and GSE NPs (50 mg/kg)-AMK group VI; (**B**–**E**) small-sized glomeruli (**G**) with widened Bowman’s spaces (**BS**), necrotic proximal tubules (**P**), marked interstitial inflammatory infiltrate (black arrow) in untreated AMK-group II, GSE (50 mg/kg)-AMK group III, GSE (100 mg/kg)-AMK group IV and GSE NPS (25 mg/kg)-AMK group V (H&E X 400)

Negative control group I showed average renal capsule, average glomeruli with average Bowman’s spaces, proximal tubules with average epithelial lining, average distal tubules and average interstitium. AMK group II showed small-sized glomeruli with widened Bowman’s spaces and markedly necrotic proximal tubules. GSE (either 50 or 100 mg/kg) treatment did not improve the hitopathological alterations that results from AMK administration; on the other hand, 50 mg/kg of GSE NPs enhanced the rats’ kidney. Where, no difference was observed in kidneys sections of both group I and VI (Fig. 7).

## Discussion

Numerous distinct antibiotics in the aminoglycoside family have been approved for use in hospitals and other healthcare facilities by the US Food and Drug Administration (FDA), including gentamicin, AMK, plazomicin, streptomycin, neomycin and paromomycin. Aminoglycosides have adverse side effects on kidney (damage both the glomerulus and the nephron). Studies on the ultrastructure of the glomerulus have provided new information about possible causes of reduced glomerular filtration (Cojocel et al. 1984). Aminoglycosides exposure changes the shape of the glomerulus, reduces the quantity and size of fenestrae, increases the thickness of the basement membrane, and reduces the surface area accessible for ultrafiltration in the glomerulus (Prayle et al. 2010). One of the aminoglycoside antibiotics, AMK, inhibits protein production in gram-negative and some gram-positive bacteria. The wider antimicrobial range of AMK and its ability to resist enzymes’ degradation (that inactivate other aminoglycosides) are its most distinctive features. As a result, AMK is useful for managing fevers and other systemic ailments in plasmid-mediated tolerance bacteria. Because of its possible nephrotoxicity, long-term use of AMK is not advised (Dwivedi et al. 2009). The reduction of AMK-induced nephrotoxicity permits its continued administration, reduces its adverse effects, and diminishes tissue injury. Numerous studies have been done on the causes of aminoglycoside poisoning (Lin et al. 2011). The generation of oxygen free radicals and an increase in cellular oxidative damage are the two potential processes by which AMK-induced nephrotoxicity is brought about. Prayle et al. (2010) reported serious cellular damages and apoptosis activation, after aminoglycosides uptake, which led to loss of epithelial layers of renal tubules. Xu et al. (2008) showed that proteinuria and the urinary loss of brush border enzymes were an indication of dysfunction of proximal tubules, disturbance in membranes’ transporters production, reduced formation of parathyroid hormone-induced cAMP and uptake of magnesium (Kang et al. 2000).

Over the past ten years, a lot of work has focused on preventing or reducing the effects of aminoglycoside nephrotoxicity. The recommended dosage should be carefully followed, serum levels should be monitored, and the buildup of aminoglycosides by the kidneys should be reduced or avoided. Two methods have been used to reduce the uptake: 1- complexing aminoglycosides extracellularly; and 2- reducing or interfering with drug attachment to the brush-border membranes. However, the approach based on the binding competition ultimately led to the discovery that aminoglycoside might be its own competitor. According to Smyth and Tan (2017), one dose/day would be anticipated to be less nephrotoxic than many divided daily doses. The drug’s kidney clearance varies throughout the day, with nighttime clearance being lower. If the aminoglycoside is taken at night instead of in the morning, the kidneys would be exposed to it more frequently (Touw et al. 2007).

Inflammation, fibrosis, and oxidative stress are the molecular processes that are extensively reported in the aetiology of CKD (Levey and Coresh 2012). Numerous plants extracts have long been known to reduce renal failure by acting as antioxidants, which has positive effects on fibrosis and inflammation (Wang et al. 2022). Preclinical study based on plants treatments, in vitro and in vivo, showed some significant therapeutic advantages (Gobe and Wojcikowski 2019). However, the administration of plant extracts in conjunction with widely accepted conventional medications may have combined or synergistic renal protective benefits (Khan et al. 2022). GSE has been reported to contain a polyphenolic compounds that exhibited antioxidant and anti-inflammatory effects (This et al. 2006). Turki et al. (2016) reported that GSE enhanced proteinuria and glomerular filtration rate (GFR), raised the anti-oxidant state as measured by the elevated plasma SOD levels and decreased carbonylation and lipoperoxidation. And added that GSE reduced inflammation by lowering C-reactive protein (CRP) and cholesterol levels as well as combating anaemia and thrombocytopenia. They concluded that some renal function parameters in CKD patients were enhanced by receiving two g GSE/day for a six-month period, and this beneficial impact of GSE appears to be at least partially mediated by its anti-inflammatory and antioxidant characteristics. In haemodialysis patients, supplementing their diets with concentrated red grape juice has been shown to have antioxidant and anti-inflammatory effects (Castilla et al. 2008). Recent research on animals showed that grape seed polyphenols can help to treat kidney failure (Gao et al. 2014; Bao et al. 2015). Terra et al. (2009) showed that high fat diet complemented with grape seed procyanidins downregulated CRP mRNA and reduced both of TNF-α and IL-6 levels in rats.

Although GSE is frequently used as a dietary supplements, excessive intake of it result in some unfavourable effects such as hemorrhage, nausea, indigestion, headache, cough, allergy, and sore throat (Berry et al. 2016). It impacts the action of some drugs as blood thinners and combine with other medications (Berry et al. 2016). Recent studies have demonstrated that large doses of GSE (greater than 500 mg/kg) have significant, positive results, whereas smaller dosages were less effective (Tsao et al. 2012; Yang et al. 2012; Goey et al. 2013; Jhun et al. 2013). NPs have a number of advantages, such as a long lifetime, the capacity to integrate both water-friendly and water-resistant substances, and the ability to be given in a number of ways (including oral application and inhalation); in addition to the regulated drug release.

This study aimed to synthesize novel GSE NPs to be used as a renoprotective agent against AMK. Grape seeds were extracted by ethyl alcohol according to the methods of Farid et al. (2022). Before the synthesis of NPs, GSE was characterized to identify the bioactive compounds by GC-MC and HPLC techniques. By using GC-MS, terpenoid alcohols like geraniol, borneol, linalool, and -terpeniol were found in GSE. Aldehydes like the benzoic aldehyde and phenolic acids like gallic and egallic acids, which are typically found in grape seeds, were also found. The results showed that there are several groups of flavonoids, such as flavanols (catechine and epicatechine), flavonols (Kaempferol, Myricetin and quercetin). Moreover, gallic acid, coffeic acid, catechin, and epicatechin were the main compounds found in GSE, according to the HPLC findings, in addition to procyanidins. According to the ICP results, calcium and potassium both had the greatest elemental concentrations.

Our results were similar to those of Al-fekaiki and Ali (2016) who reported that oleic acid made up 16.98% of the fatty acids in GSE, followed by palmitic acid, stearic acid, palmitoleic acid, myristic acid, azelaaldehydic acid, margaric acid and lauric acid, pentadecanoic acid (12.09, 8.85, 0.28, 0.21, 0.12, 0.11, 0.05 and 0.03%, respectively). In addition, Gorodyska et al. (2018) reported that the obtained data from GC-MS proved the presence of a significant amount of phenol compounds from various sources in GSE, including resveratrol, flavonoids like quercetin and its derivatives, catechine, epicatechine, and campferol, as well as esters of fatty acids. Grases et al. (2015) reported that the major phenolic compounds (identified by HPLC) in GSE was epicatechin, catechin and procyanidin dimers (B1-B4) and the procyanidin trimer. Also, Simonetti et al. (2020) reported that the phenolic compounds in GSE were represented primarily by gallic acid. Ignat et al. (2011) said that gallic acid and catechin was the major phenolic compounds in GSE. According to results of Ozcan (2010), high concentrations of calcium, potassium, magnesium, sodium, phosphors and sulphur were found in GSE. In addition, aluminum, iron, manganese and zinc were found by low concentration. Toaldo et al. (2013) found the elements copper, nickel, barium and aluminum in GSE.

After identification of the bioactive compounds in GSE, the extract powder was transformed into NPs. Where, GSE powder was mixed with HCl (30%) and stirred for 60 min (at 30° C and 3000 rpm), followed by the addition of 100 ml distilled water. The solution was stirred for an additional two hours, followed by filtration to recover GSE NPs. The formed NPs were characterized by electron microscope to determine its shape and size, where, GSE NPs have a smooth surface and a size that ranged from 40 to 70 nm. The GSE NPs showed the highest DPPH scavenging % at all tested concentration (1000, 500, 125, 62.5 and 31.25 µg/ml) when compared to ascorbic acid (control). Moreover, the antioxidant activity of GSE NPs was increasing with the elevation in concentration; where, 96% were achieved with 1000 µg/ml of GSE NPs. A direct relation between the concentration of GSE or GSE NPs and the clotting time was observed; however, the prepared NPs have a lower clotting time than that of heparin. Therefore, it can be used in vivo safely without the fear of inducing hemorrhage. Also, GSE NPs inhibited the hemolysis of RBCs by 93.1% indicating a marked anti-inflammatory effects; and showed an anti-cytotoxic effects indicating its safety to be used in vivo. In addition, GSE NPs showed a strong antimicrobial activity against *Bacillus subtilis*, *Staphylococcus aureus*, *Escherichia coli*, *Pseudomonas aeruginosa*, *Candida albicans* and *Mucor*.

The in vivo testing revealed that GSE NPs significantly reduced the elevated levels of BUN, urea, creatinine and urinary albumin (due to AMK administration). The antioxidant and anti-inflammatory effects (in vivo) of GSE NPs were proofed from the reduction of lipid peroxidation (MDA) and pro-inflammatory cytokines (TNF-α, IL-4 and IL-6) levels; and the elevation of the antioxidant enzyme (SOD) level. Moreover, the histopathological alterations, in kidney sections, that results from AMK administration were enhanced in rats’ groups receiving GSE NPs (50 mg/kg). The experimental groups showed that 50 mg/kg of GSE NPs has a renoprotective effect more than 100 mg/kg of GSE; this means GSE NPs can be used by low doses to avoid the adverse effect of GSE.

Our results can be explained by: 1- GSE NPs were rich in polyphenol compounds like proanthocyanidins, phenolic acids like gallic and egallic acids, flavanols (catechine and epicatechine), flavonols (Kaempferol, Myricetin and quercetin), 2- these bioactive compounds have a strong antioxidant activity and scavenge ROS, control immune function and platelet activation (Varzakas et al. 2016), 3- GSE NPs reduced inflammation that followed AMK administration and 4- GSE NPs attenuated the AMK-induced nephrotoxicity and protected the rats’ kidney.

GSE NPs have an anti-oxidant, anti-inflammatory, anti-cytotoxic and anti-microbial in vitro effects. It is safe to be used in vivo as a renoprotective agent against AMK; where, it reduced the oxidative stress and inflammation.

## Data Availability

All data generated or analysed during this study are included in this published article.
